# Astrocytes in Oligodendrocyte Lineage Development and White Matter Pathology

**DOI:** 10.3389/fncel.2016.00119

**Published:** 2016-05-10

**Authors:** Jiasi Li, Lei Zhang, Yongxin Chu, Michael Namaka, Benqiang Deng, Jiming Kong, Xiaoying Bi

**Affiliations:** ^1^Department of Neurology, Shanghai Changhai HospitalShanghai, China; ^2^Department of Vascular Surgery, Shanghai Changhai HospitalShanghai, China; ^3^Department of Vascular Surgery, Affiliated Huai’an Hospital of Xuzhou Medical CollegeHuai’an, China; ^4^Faculty of Health Sciences, College of Pharmacy and Medicine, University of ManitobaWinnipeg, MB, Canada; ^5^Department of Human Anatomy and Cell Science, University of ManitobaWinnipeg, MB, Canada

**Keywords:** astrocyte, white matter, oligodendrocyte, demyelination, neurodegeneration, psychiatric disorders

## Abstract

White matter is primarily composed of myelin and myelinated axons. Structural and functional completeness of myelin is critical for the reliable and efficient transmission of information. White matter injury has been associated with the development of many demyelinating diseases. Despite a variety of scientific advances aimed at promoting re-myelination, their benefit has proven at best to be marginal. Research suggests that the failure of the re-myelination process may be the result of an unfavorable microenvironment. Astrocytes, are the most ample and diverse type of glial cells in central nervous system (CNS) which display multiple functions for the cells of the oligodendrocytes lineage. As such, much attention has recently been drawn to astrocyte function in terms of white matter myelin repair. They are different in white matter from those in gray matter in specific regards to development, morphology, location, protein expression and other supportive functions. During the process of demyelination and re-myelination, the functions of astrocytes are dynamic in that they are able to change functions in accordance to different time points, triggers or reactive pathways resulting in vastly different biologic effects. They have pivotal effects on oligodendrocytes and other cell types in the oligodendrocyte lineage by serving as an energy supplier, a participant of immunological and inflammatory functions, a source of trophic factors and iron and a sustainer of homeostasis. Astrocytic impairment has been shown to be directly linked to the development of neuromyelities optica (NMO). In addition, astroctyes have also been implicated in other white matter conditions such as psychiatric disorders and neurodegenerative diseases such as Alzheimer’s disease (AD), multiple sclerosis (MS) and amyotrophic lateral sclerosis (ALS). Inhibiting specifically detrimental signaling pathways in astrocytes while preserving their beneficial functions may be a promising approach for remyelination strategies. As such, the ability to manipulate astrocyte function represents a novel therapeutic approach that can repair the damaged myelin that is known to occur in a variety of white matter-related disorders.

## Introduction

Human white matter makes up approximately half of the forebrain volume, which is 3–4 times higher than that in rodents (Hamner et al., 2011). White matter in the mammalian central nervous system (CNS) is primarily composed of myelin and myelinated axons. Structural and functional completeness of myelin is critical for the reliable and efficient transmission of information. White matter damage caused by ischemia, hypoxia, inflammation and trauma has been associated with the development of primary or secondary demyelinating diseases. Demyelination of white matter is directly correlated with a vast array of clinical symptoms such as but not limited to sensory disturbances, paralysis, psychiatric disorders and cognitive impairments. Despite the variety of scientific advances that have been developed to promote myelin repair, unfortunately, at best they have only had marginal success. Based on the scientific literature, we do know that cells of the oligodendrocyte lineage are responsible for myelinating CNS axons. Oligodendrocyte precursor cells (OPCs) represent the major source of cells that are required to differentiate into mature oligodendrocytes. Oligodendrocytes are responsible for myelination during embryonic development and after a CNS demyelinating event (Crawford et al., 2014). The inability to induce the required production of OPCs is thought to occur due to the failure of the microenvironment to support their regeneration (Fancy et al., 2014).

In the CNS, astrocytes comprise the most ample and diverse type of glial cells found in both white matter and gray matter. They display many essential and complex functions in adult brain and spinal cord. Specifically they are involved in participation and regulation of synaptogenesis (Clarke and Barres, 2013), support and nourishment of ambient parenchymal cells (Allaman et al., 2011), homeostasis maintenance of water, ions and neurotransmitters (Obara et al., 2008; Papadopoulos and Verkman, 2013), development and maintenance of the vasculature scaffold and blood-brain barrier (BBB), as well as modulation of local blood flow (Attwell et al., 2010). As a result, astrocytes represent a very unique cell type that can directly influence the production and survival of cells of the oligodendrocyte lineage.

Astrocytes respond to multiple forms of CNS insult, such as trauma, ischemia, infection, inflammation, neurodegeneration etc. They become “active” or “reactive” with the changes in their morphology, physiology and gene expression (Sofroniew and Vinters, 2010). Research suggests that reactive astrocytes are considered detrimental to myelin repair or the re-myelination process because of their involvement in producing scar tissue and inhibiting OPC survival, differentiation, migration, and axonal connectivity (Wang et al., 2011). However, in recent years, there is accumulating evidence to suggest that astrocytes can also create a permissive environment for oligodendrocyte lineage expansion and differentiation. They demarcate and separate the injured area to limit the spread of toxic factors (Fitch and Silver, 1997) and provide tropic factor to survive the surrounding cells of lesions (do Carmo Cunha et al., 2007). Removal of reactive astrocytes after CNS injury led to larger lesions, severe demyelination and oligodendrocyte death (Sofroniew and Vinters, 2010; Haroon et al., 2011). Glial Fibrillary Acidic Protein (GFAP) knockout mice revealed more extensive abnormalities in the white matter than in the gray matter (Liedtke et al., 1996).

During development and regeneration, cells from the oligodendrocyte lineage keep in intimate contact with astrocytes that regulate their activity and function (Moore et al., 2011). As such, extensive research has recognized the importance of astrocytes in the modulation of oligodendrocyte proliferation and differentiation (Noble et al., 1988). An increasing wealth of scientific evidence suggests that the astrocytic response determines whether white matter lesions are able to re-myelinate (Barnett and Linington, 2013). Irrespectively, the exact mechanisms by which they affect re-myelination still remain elusive. In this review, we will highlight the features of astrocytes in CNS white matter and address the versatile roles by which astrocytes affect OPCs and/or oligodendrocytes, ultimately determining the degree of myelin repair or re-myelination that occurs following white matter damage.

## Astrocytes in White Matter

Astrocytes are heterogeneous in many aspects, such as development, morphology, location, protein expression and function. They are mainly derived from radial glia in developmental brain and are generated by subventricular stem cells in the adult brain (Tsai et al., 2012). A bipotential progenitor cell population that expresses A2B5 surface marker also can differentiate into either type 2 astrocytes or oligodendrocytes *in vitro* (Raff et al., 1983). The astrocytes originating from different progenitor cells stay in place and are not replenished by those in neighboring domains (Tsai et al., 2012). Astroglial morphology, density and proliferation independently define the discrete cytoarchitecture of the adult mammalian CNS (Emsley and Macklis, 2006).

Astrocytes in gray matter are typically larger than those in white matter. The main type of astrocytes in gray matter is the protoplasmic astrocyte (designed as type-1 astrocytes), which are bushy or spongiform and possesses numerous highly branched fine processes that spread more or less radially from the soma. At least one of the processes contacts blood vessels *via* perivascular endfeet. In white matter there mainly are the fibrous astrocytes (designed as type-2 astrocytes), which have less branched and thicker processes with smooth, rough, straight, or undulating appearance and much longer length than protoplasmic astrocytes (Sun et al., 2009). The somas of fibrous astrocytes are often evenly spaced and ranked in rows between the axon bundles, and their processes terminate at nodes of Ranvier, the sites of action potential generation (Butt, 2011).

Neighboring protoplasmic astrocytes have non-overlapping spatial domains with little overlap of neighboring processes (Halassa et al., 2007). They are most evident in the areas of high synaptic density, indicating main participation of astrocytes in modulating synaptic activities in gray matter. Fibrous astrocytes do not have individual spatial domains and the processes of neighboring astrocytes overlap extensively, suggesting their supporting or metabolic effects in white matter (Sun and Jakobs, 2012).

Astrocytic densities and proliferation rates vary broadly in different white matter tracts. The density of fibrous astrocytes ranges from virtually zero in the stria terminalis to 100–125 cells/mm^2^ in the optic tract. Corpus callosum, the largest fiber tract in white matter, contains 79 ± 4 GFAP-positive cells/mm^2^. Dentate gyrus has the highest proliferation rate of astrocytes across the adult mammalian CNS, followed by subventricular zone and rostral migratory stream when estimated with bromodeoxuridine (BrdU)/GFAP-positive cells (Emsley and Macklis, 2006).

Astrocytes express intermediate filament proteins depending on their types and developmental stages. GFAP is a kind of characteristic biomarker which is expressed exclusively by astrocytes in the CNS. Up-regulation of GFAP expression under pathophysiological conditions is generally considered as a hallmark of reactive astrocytes (Sofroniew and Vinters, 2010). GFAP, vimentin and nestin are highly expressed in white matter compared to gray matter, so are glutamate-aspartate transporter (GLAST), glutamate transporter-1 (GLT-1) and glutamine synthetase (GS; Goursaud et al., 2009). Elevated GFAP expression may help GLAST anchored in the membrane of reactive astrocytes (Sullivan et al., 2007), indicating astrocytes in white matter have more powerful capacity of glutamate clearance than those in gray matter. The result is in accordance with the metabolic features of oligodendrocyte lineage. Oligodendrocytes eventually have to support membranes up to 100 times the weight of their cell body, which consequently leads to extremely high metabolic rates with the production of many toxic byproducts. However, cells of the oligodendrocyte lineage have only low concentration of the anti-oxidative enzyme glutathione. Effective glutamate clearance and release of glutathione by astrocytes are the major mechanisms preventing accumulation of glutamate and maintaining healthy oligodendrocytes (Kimelberg and Nedergaard, 2010).

## The Effect of Astrocytic Roles on Oligodendrocyte Lineage

### An Energy Supplier

The adult brain occupies only 2% weight of our body but consumes nearly 20% of glucose and oxygen. There is no direct glucose storage in our brain cells. Under normal physiological conditions, astrocytes and oligodendrocytes take in glucose directly from the surrounding blood vessels through glucose transporter 1(GLUT1). However, during hypoglycaemia or aglycaemia, as well as during the period of increased neural activities, astrocyte-derived glycogen is the main energy source (Brown et al., 2003). Astrocytes are the sole repository for glycogen particles in the mammalian CNS because glycogenesis is confined only to astrocytes (Cataldo and Broadwell, 1986) and also because astrocytic endfeet cover more than 90% of the cerebral vasculate, which makes them absolutely dominant to obtain the glucose. As such, they are the storage site of glycogen (Baltan, 2015).

Astrocytic glycogen is released in the form of lactate by glycolysis on demand, and lactate is transported through monocarboxylate transporter (MCT) to ambient parenchymal cells. Glial cells express both MCT1 and MCT4. MCT1 expression is much more pronounced in oligodendrocytes than astrocytes (Baltan, 2015). As such, this suggests that oligodendrocytes need much lactate *via* MCT1 from both astrocytes and blood circulation as a source of energy and a precursor of lipids to build carbon skeletons. The oxidative rate of lactate is higher and the rate of lipid synthesis from lactate is at least six-fold higher in oligodendrocytes than those in astrocytes or neurons (Sánchez-Abarca et al., 2001; Rinholm et al., 2011). Oligodendrocytes actively utilize lactate by oxidation and lipogenesis which are required to synthesize myelin, a specialized lipid—rich membrane. It has been found that oligodendrocytes are more vulnerable to ischemia or hypoxia than neurons (Pantoni et al., 1996). This may be one of the susceptible reasons that limit their ability to re-myelinate or repair damaged areas of myelin. Fibrous astrocytes in white matter demonstrate very high rates of glucose oxidation through the pyruvate dehydrogenase-catalyzed reaction and through the tricarboxylic acid cycle, while protoplasmic astrocytes in gray matter show high biosynthetic activity (Sánchez-Abarca et al., 2001). However, it is unclear if the priority of astrocytic functions is corresponding to their physiological actions in different regions and as such, this requires further investigative research.

When glucose levels are low, L-lactate, rather than D-lactate is responsible for protecting oligodendrocytes and OPCs and as such rescues myelination from the reduction (Brown et al., 2003; Rinholm et al., 2011). When glucose is sufficient, lactate can also be preferred over glucose as a substrate for myelin production (Rinholm et al., 2011; Baltan, 2015), implying oligodendrocyte lineage may need additional energy and carbon sources even in normal glucose levels. In 1994, the hypothesis of astrocyte—neuron lactate transfer shuttle (ANLTS) was proposed, stating lactate released from astrocytes through glycolysis could be directed one-way traffic *via* MCTs from astrocytes to neurons (Pellerin and Magistretti, 1994). This begged the question as to whether there is a similar “lactate shuttle” in white matter. Astrocytes can deliver lactate to axons at the site of nodes of Ranvier (Baltan, 2015). It has been found that oligodendrocytes *in vitro* and *in vivo* don’t consume but rather are a potential source of lactate to support axon function. These findings indicate or suggest the involvement of a myelin—independent mechanism of axon loss (Funfschilling et al., 2012). Whether there is lactate shuttle between astrocytes and oligodendrocytes and how they inter-coordinate to supply the lactate to axons are worthy of much attention.

### A Participant of Immunological and Inflammatory Functions

There is growing evidence that demonstrates astrocytes work as a kind of immune cells. They take part in formation of BBB, secrete cytokines and inflammatory mediators, regulate functions of microglia/macrophage cells, as well as present antigen *via* major histocompatibility complex and indirect or direct phagocytosis. Various observations strongly suggest that astrocytes participate in two-way communications with immune and inflammatory cells and thereby play an irreplaceable role in regulating CNS immune and inflammatory reactions (Hamby et al., 2012; Sofroniew, 2014), depending on different triggered signaling pathways, locations and times.

Transcription factor nuclear factor kappa B (NF-κB) appears to have an important role in understanding the inflammatory effect of astrocytes on oligodendrocytes. Transgenic technology (especially the deletion experiments) represents a new *in vivo* tool that is being used to advance our understanding of the role of NF-κB on astrocytes and oligodendrocytes. For example, a mouse model of GFAP—IκBα–dominant-negative (dn) is made by inactivating NF-κB in astrocytes through overexpression of a dn form of the NF-κB super repressor (IκBα-dn) under control of the GFAP promoter. In this model oligodendrocytes are protected* via* reduced immune-cell infiltration (Brambilla et al., 2014) and downregulation of adhesion molecules and chemokines (Brambilla et al., 2009) at acute or chronic stage of experimental autoimmune encephalomyelitis (EAE). These molecules and chemokines include macrophages/granulocytes (CD45^hi^CD11b), MHCII^+^ macrophages/granulocytes, B cells (B220), activated B cells (B220MHCII), natural killers (NK1.1), CD4 T cells, ICAM-1, VCAM-1, Itg-β_5_, Itg-β_7_, CXCL9, CXCL10 and their receptor CXCR3 etc. At 6 days after inducing re-myelination of EAE model in GFAP—IκBα–dn mice, increased oligodendrogenesis, rather than the number change of OPCs, is distributed around the epicenter. It suggests the anti-inflammatory niche near the lesion in GFAP-IκBα-dn group is more permissive for the differentiation of OPCs into mature oligodendrocytes (Brambilla et al., 2009). Conditional deletion of related factors in astrocytic NF-κB pathways, such as Act1 (Raasch et al., 2011; Kang et al., 2013b), IkB kinase 2 (Raasch et al., 2011), NEMO (van Loo et al., 2006) and so on, lead to its inactivity thereby exert protective influence on the cells of oligodendrocyte lineage through inhibiting the release of pro-inflammatory factors. Laquinimod (LAQ), a new oral immunomodulatory compound for relapsing and remitting multiple sclerosis (RRMS), can ameliorate myelin injury *via* down-regulating NF-κB activation in astrocytes rather than in microglia *in vivo* and *in vitro* (Brück et al., 2012).

Chemokines represent another well-known molecular cue expressed by astrocytes that affect oligodendrocyte biological behavior. Astrocyte-conditioned media collected from astrocytes pre-exposed to pro-inflammatory supernatants lead to reduced OPCs differentiation without increasing cell death obviously through astrocyte-derived CXCL10 (Moore et al., 2015). Deletion of astrocytic CXCL10 delay clinical onset but doesn’t affect progressive axon loss in EAE mice (Mills Ko et al., 2014). Astrocytes transiently express a high level of CXCL1 during the spinal cord development, and CXCL1-mediated signaling through its receptor CXCR2 on OPCs promote their proliferation* via* a platelet-derived growth factor (PDGF)-AA-driven mechanism and inhibit migration (Tsai et al., 2002). The mice models with conditional gene deletion of CCL2 (also as monocyte chemoattractant protein-1, MCP-1) from astrocytes have less severe EAE late in disease and slow progression of spinal cord axon loss, involving significantly decreasing accumulation of classically activated M1 microglia (Moreno et al., 2014), less macrophage and T cell inflammation (Kim et al., 2014), the lack of leukocyte penetration and disrupted claudin -5 at the BBB in CNS (Paul et al., 2014). However, the effects of CCL2 deletion in astrocytes on acute stage or onset of EAE are discrepant and warrant further researches (Kim et al., 2014; Moreno et al., 2014). (Table 1).

**Table 1:** **The effect of astrocyte-derived factors on oligodendrocyte lineage or EAE**.

Astrocyte-derived factors	Model	Effect on oligodendrocyte lineage or EAE	Reference
CXCL1	Spinal cord	Inhibiting OPCs migration and promoting OPCs proliferation	Tsai et al. (2002)
CCL2	MOG-induced EAE	Reducing acute and long-term severity of clinical deficits;	Brambilla et al. (2009); Moreno et al. (2014); and Paul et al. (2014)
		Exacerbating severity of EAE at late stage	Kim et al., 2014
CXCL10	MOG-induced EAE	Be related with clinical onset of EAE	Mills Ko et al. (2014)
IL-6	Astrocyte-conditioned media pre-exposed to the proinflammatory supernatants	Reducing OPCs differentiation without an apparent increase in cell death	Moore et al. (2015)
IL-1β	Hypoxic PWMD	Inducing oligodendrocyte apoptosis	Deng et al. (2014)
BDNF	Cuprizone-induced demyelination	Reversing deficits elicited following demyelination	Fulmer et al. (2014)
BMP	Spinal cord injury	Inhibiting OLs differentiation	Wang et al. (2011)
CNTF	Spinal cord injury	A pro-OLs differentiation and survival factor	Hesp et al. (2015)
FGF-2	Murine hepatitis virus-induced demyelination of the spinal cord	A potent mitogen for OPCs	Albrecht et al. (2003)
IGF-1	Primary cultured astrocytes	Promoting OPCs maturation	Clarner et al. (2011)
LIF	Astrocyte–oligodendrocyte coculture model	Promoting OLs Maturation	Ishibashi et al. (2006) and Fischer et al. (2014)
Neuregulin	Mice bearing a null mutation in the neuregulin gene	Be necessary for the normal development of OLs	Viehover et al. (2001)
Osteopontin	Cuprizone-induced demyelination	Inducing proliferation of OPCs	Selvaraju et al. (2004)
PDGF	Cuprizone-induced demyelination in mice	Increasing OPCs population density	Woodruff et al. (2004)
Hyaluronan	Lysolecithin or MOG-induced EAE	Inhibiting OPCs maturation	Back et al. (2005)
Fibronectin, vitronectin, and laminin combined with bFGF	Mixed culture containing human OLs, astrocytes, or microglia	Promoting process outgrowth by adult human OLs	Oh and Yong (1996)

Astrocytes also secrete cytokines directly or indirectly modulating oligodendrocyte lineage and myelination. In rat models of periventricular white matter damage (PWMD) caused by hypoxic exposure in the peri-natal period, astrocyte-derived tumor necrosis factor (TNF)-α and interleukin (IL)-1β induce apoptosis of oligodendrocytes (Deng et al., 2014). However, elective stimulation of human TNF recptor2 (TNFR2) on astrocytes can promote OPCs differentiation into mature oligodendrocytes by secretion of leukemia inhibitory factor (LIF) *via* PI3K-PKB/Akt pathway (Fischer et al., 2014). Interferon (IFN)-γ functions as a pro-inflammatory cytokine at the early stage of EAE. However, in transgenic mice expressing a signaling deficient dominant negative IFN-γ receptor 1 specifically on astrocytes (GFAPγR1△ mice), IFN-γ signaling to astrocytes limits demyelination during acute EAE and regulates inflammation, thus promoting remission (Hindinger et al., 2012), indicating astrocytes participate in limiting CNS autoimmune disease dependent upon a neuroprotective signaling pathway *via* engagement of IFN-γ receptors. Transforming growth factor (TGF) β1-induced activation of Jagged1-Notch1 signaling in astrocytes via ALK5 and Smad3 may impact the size and differentiation of the OPC pool in the human CNS (Zhang et al., 2010). Taken together, astrocyte-derived immunological and inflammatory factors have variable effects on oligodendrocyte lineage in context-specific manners.

### A Source of Trophic Factors and Iron

Astrocytes secrete many tropic factors which affect oligodendrocyte lineage during development and remyelination and are an indispensable part of astrocytic anti-inflammatory function. Astrocytes are the main producer of PDGF (Kernt et al., 2010) which could be enhanced by IFN-γ, IL-5 and IL-17 (Moore et al., 2015) in CNS, suggesting the secretion of astrocyte-derived PDGF can be regulated by inflammatory stimulations. PDGF is an important survival factor for OPCs and stimulates OPCs to either proliferation or differentiate into mature oligodendrocytes, depending on the developmental stage of the progenitor cells (Gard et al., 1995). Although astrocyte-derived PDGF increases OPCs density either in development or in focal demyelinated lesions, there is no difference in the time course or extent of remyelination between PDGF-increased group and wild-type group (Woodruff et al., 2004), indicating the availability of OPCs isn’t the critical limitation of remyelination.

Fibroblast growth factor-2 (FGF-2), expressed by both neurons and glia during development, mainly comes from astrocytes after myelin injury (Messersmith et al., 2000). It is a potent mitogen for OPCs and most of the time, related to successful re-myelination (Messersmith et al., 2000; Albrecht et al., 2003; Hesp et al., 2015). However, application of FGF-2 sometimes becomes a handicap for myelin repair. FGF-2 in combination with PDGF promotes OPCs renewal but prevents their differentiation (Clarner et al., 2011; Ogata et al., 2011). Elevated FGF-2 leads to severe disruption of mature oligodendrocytes and a marked loss of myelin (Butt and Dinsdale, 2005) or even converts mature oligodendrocytes to a novel phenotype (Bansal and Pfeiffer, 1997). How astrocytic FGF-2 exerts its influence hinges on the status of oligodendrocyte lineage.

Ciliary neurotrophic factor (CNTF) is produced by astrocytes and neurons in the CNS. It is an important survival factor for oligodendrocytes, promotes OPCs differentiation into mature myelin-forming cells, helps differentiated oligodendrocytes to synthesize myelin and protects oligodendrocytes from inflammatory insult (Stankoff et al., 2002; Modi et al., 2013). Insulin-like growth factor-1 (IGF-1) is widely expressed in the CNS and its expression in astrocytes is increased during and after variety of CNS injuries (Lee et al., 1996). Astrocytic IGF-1 promotes significant increase in the number of oligodendrocytes and myelin *in vivo* by directly affecting IGF type 1 receptor (IGF1R) signaling in the cells of oligodendrocyte lineage during normal oligodendrocyte development and myelination (Zeger et al., 2007). IGF also contributes OPCs survival and augments differentiation (Zumkeller, 1997). Leukemia inhibitory factor (LIF) expressed by astrocytes is low or undetectable in normal adult tissue (Aloisi et al., 1994). It is increased by TNFR2-mediated activation of the PI3K-PKB/Akt pathway in primary astrocytes, promoting OPCs differentiation into mature myelinating oligodendrocytes (Fischer et al., 2014). Astrocytes release LIF in response to ATP liberated from axons firing action potentials, which promotes myelination by mature oligodendrocytes (Ishibashi et al., 2006). However, LIF is not always beneficial for oligodendrocyte lineage. It inhibits myelination in a manner of concentration-dependent biological activity (Ishibashi et al., 2006; Fischer et al., 2014).

There are many other trophic factors affecting myelination secreted by astrocytes, including bone morphogenetic proteins (BMP; Wang et al., 2011), neuregulin (Viehover et al., 2001), brain-derived neurotrophic factor (BDNF; Fulmer et al., 2014), osteopontin (Selvaraju et al., 2004) as well as others (as summarized in Table 1). Different trophic factors from astrocytes exert influence on myelination and repair directly or indirectly *via* autocrine or paracrine.

**Figure 1:**
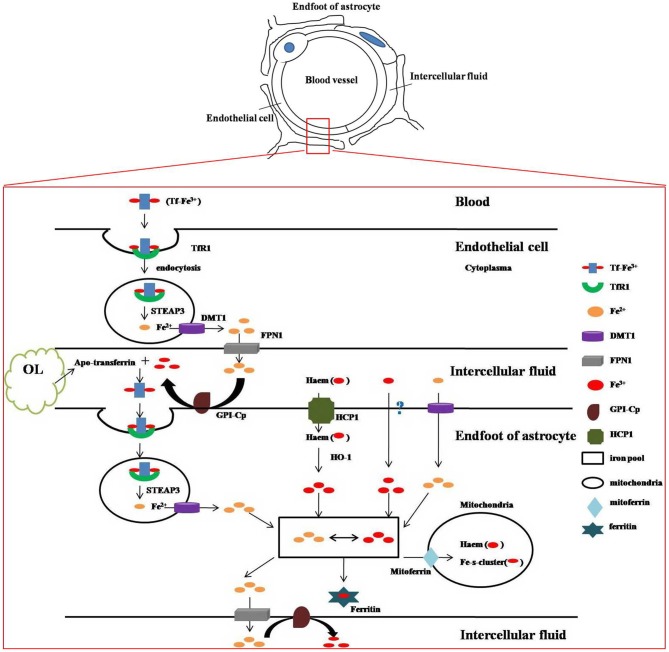
**Iron metabolism and transport in blood-brain barrier (BBB).** The above sketch demonstrates ultrastructure of BBB. The bottom figure shows how iron metabolizes and is transported between the structures of BBB. Two atoms of ferric iron (Fe^3+^) are bound to one transferrin (Tf) molecule to generate holo-transferrin, which is the main form of iron in the systemic bloodstream. Holo-transferrin interacts with surface cellular transferrin receptor 1 (TfR1) and then enters the cytosol through endocytosis. STEAP3, the endosomal metalloreductase, reduces insoluble Fe^3+^ to soluble ferrous iron (Fe^2+^) which is released into cytoplasma via divalent metal transporter 1(DMT1) and then into intercellular fluid mediated by ferroportin 1 (FPN1). Fe^2+^ is converted to Fe^3+^ by the enzyme of ceruloplasmin (CP), a kind of ferroxidase, which is attached to the membrane by glycosyl phosphatidylinisotol (GPI). Fe^3+^ combines with apo-transferrin (iron-free transferrin) which is mainly synthesized and secreted by oligodendrocytes and cells of the choroid plexus, and then enters the endfeet of astrocytes by endocytosis. In endosome Fe^3+^ is also reduced to Fe^2+^ by STEAP3 and then released to the astrocytic cytoplasma. In addition, astrocytes are also able to obtain iron from other sources. Haem-bound Fe^3+^ uptake is mediated by heme carrier protein 1(HCP1) and Fe^3+^ is liberated from internalized heme by heme oxygenase-1(HO-1). Fe^3+^ can be taken up by a kind of unknown Fe^3+^ transporter. Fe^2+^ can be transported into astrocytes via DMT1 directly. Fe^3+^ and Fe^2+^ transform into each other as needed in astrocytic cytoplasma. They may be released to outside of the astrocytes by FPN1 (Fe^2+^), stored in ferritin (Fe^3+^) or enter the mitochondria by mitoferrin which are the mitochondrial iron transporters, where iron can be used for incorporation into heme groups and iron-sulfur (Fe–S) clusters.

Iron is indispensable in mammalian metabolism because it acts as a co-factor in many metabolic reactions and participates in the formation of heme and iron-sulfur clusters. However, most of brain cells can’t get iron directly from the blood circulation because of the existence of BBB. Astrocytes are considered as a key regulator of iron in the CNS due to their extensively distributed endfeet, which almost completely covers the brain capillaries which have overwhelming location advantages to acquire iron. Numerous receptors and transporters are involved in the uptake, distribution and release of iron. Through these mediators, holo-transferrin that is the main form of iron in the systemic bloodstream enters the cytosol through endocytosis and is released in the form of ferrous iron (Fe^2+^) into cytoplasma and then into intercellular fluid. Fe^2+^ is converted to Fe^3+^ by the enzymes on the surface of astrocytes and then binds to apo-transferrin (that is, iron-free transferrin) mainly synthesized and secreted by oligodendrocytes and cells of the choroid plexus (Zakin et al., 2002). In addition, astrocytes have been shown to accumulate non-transferrin-bound iron (NTBI) from Fe^2+^ (Zakin et al., 2002; Tulpule et al., 2010) and Fe^3+^ (Philpott, 2012; Figure 1).

Of all the cell types in the brain, oligodendrocytes contain the highest level of immobilized, protein-bound iron (Todorich et al., 2009). During development, a peak period of iron influx into the brain coincides with the time point of the maximum amount of myelin production (Crowe and Morgan, 1992). However, iron may affect oligodendrocyte development during early more than late embryogenesis (Morath and Mayer-Pröschel, 2001). In adult brain, oligodendrocytes have high demand for iron reflected by their high levels of ferritin and basic requirement for their functions, oxidative metabolism and remyelination (Todorich et al., 2009). Oligodendrocytes are unable to synthesize iron, so they must obtain it from the ambient environment.

Ferritin is the main storage form of iron in the CNS. Microglia exhibits stronger stain for ferritin (Connor et al., 1990) while astrocytes express low ferritin level (Irace et al., 2005) under physiological conditions, indicating astrocytes are vital for the iron transportation, not the storage. As a kind of cells connecting directly with circulating iron, the damage to any part of astrocytic iron transportation may have an influence on oligodendrocyte lineage. The astrocyte-specific FPN knockout demyelinating-mouse induced by lysolecithin microinjection produces a clear decrease in proliferation of OPCs, leading to reduced remyelination. Lack of iron reduces the secretion of pro-inflammatory cytokines (i.e., IL-1 and TNF-α) from microglia, therefore resulting in down-regulation of FGF-2 and IGF-1 generated by IL-1-stimulating and TNF-α-stimulating astrocytes, respectively (Schulz et al., 2012). Furthermore, even though iron is supplemented after a period of iron-deprivation to the normal serum level, morphological damage of myelin from iron-deficiency is not easily reverted by iron reposition (DeMaman et al., 2008). In a demyelinating rat induced by myelin mutant, iron accumulation, expression of ferritin and antioxidant enzyme heme oxygenase-1 (HO-1) in astrocytes are increased, indicating a protective reaction of astrocytes to alleviate iron-mediated cytotoxicity by regulating autologous iron-related pathway (Izawa et al., 2010). Taken together, astrocytes act as a direct gainer of iron in the CNS and demonstrate pivotal effects on oligodendrocyte lineage with high demand for iron.

### A Sustainer of Homeostasis

Oligodendrocyte lineage is highly vulnerable to the excitatory neurotransmitter glutamate because of abundant glutamate receptors on cell surface and low content of reduced glutathione (Sánchez-Gómez et al., 2011). Astrocytes express high levels of glutamate transporters-excitatory amino acid transporters 1 (EAAT1, also as GLAST) and EAAT2 (also as GLT-1). Astrocytic uptake of glutamate is the major mechanism preventing accumulation of glutamate in the extracellular space and thus protecting oligodendrocyte lineage from excessive activation and excitotoxic cell death. They take in glutamate and release glutamine (glutamate-glutamine cycle), take in glutathione precursors and release glutathione (Kimelberg and Nedergaard, 2010). The loss of glial GLAST or GLT-1 produces elevated extracellular glutamate levels. Decreased uptake of glutamate by astroglial EAATs causes oligodendroglia death in hypoxic PWMD (Murugan et al., 2013). The activation of mammalian target of rapamycin (mTOR)-Akt-NF-γB signaling cascade, which promotes glutamate uptake by up-regulation of astrocytic GLT-1, may be potential protection (Ji et al., 2013). In addition, glutamine is an important component of proteins and an excellent energy substrate. However, oligodendrocytes do not have GS and require glutamine from astrocytes.

Astrocytes are a key regulator of ions and water producing a permissive microenvironment for development and survival of oligodendrocyte lineage. Physical contacts of gap junctions between astrocytes and oligodendrocytes form panglia syncytium supporting a unidirectional flow of cytosolic contents from astrocytes to oligodendrocytes. The major function of this coupling is to provide potentially K^+^ spatial buffering and serves to dissipate the increased K^+^ concentration in oligodendrocytes* via* direct flow into astrocytic cytoplasm (Kofuji and Newman, 2004). Na^+^/H^+^ exchanger (NHE) is the most important plasma membrane transporter involved in the astrocytic pH regulation. Blocking NHE1 activity by the potent NHE1 inhibitor HOE-642 significantly reduces the resting level of intracellular pH in NHE1^−/−^ astrocytes (Kintner et al., 2004). After hypoxia or ischemia, NHE-1 protein is up-regulated in astrocytes and inhibition of NHE-1 is neuroprotective by ameliorating disruption of ionic homeostasis (Cengiz et al., 2011).

All transmembrane ionic shifts and membrane transport mechanisms such as glutamate uptake are followed by water movement. Unbalance of the level of extracellular ions and neurotransmitters activates astrocytic regulatory volume changes, resulting in the shrinkage or swelling of astrocytes (Benesova et al., 2009). Aquaporin4 (AQP4) water channels expressed in the perivascular end-feet of astrocytes contribute to water homeostasis by regulating water transport and are involved in several disease pathways (Amiry-Moghaddam et al., 2003). In an ischemic stroke model, the presence of AQP4 was shown to aggravate post-ischemic cytotoxic edema, while AQP4-KO mice demonstrated an improved neurological outcome (Wang W. W. et al., 2014). Neuromyelities optica (NMO) is a representative condition of inflammatory demyelinating disorder caused by targeting astrocytic AQP4. As such, this indicates that direct damage to astrocytes can influence the integrity of oligodendrocyte-myelin unit.

Many studies have shown how astrocyte-secreted extracellular matrix (ECM) affects the behavior of oligodencrocyte lineage, particularly OPCs. Astrocyte-derived hyaluronan may inhibit OPC maturation (Back et al., 2005), leading to remyelination failure. However, astrocytic ECM containing fibronectin, vitronectin, and laminin combined with bFGF is found to promote oligodendroglial process extension *in vitro* (Oh and Yong, 1996). Astrocytic secretion changes the components of ECM, thereby adjusts a favorable or adverse surrounding environment of re-myelination, depending on the protein composition and the receptors on the OPCs. In summary, astrocytes are a pivotal controller for homeostasis, thus extensively influence oligodendrocyte lineage.

## Astrocytes in White Matter Pathology

### Demyelinating Diseases

As mentioned above, NMO is a typical demyelinating disorder induced by direct autoimmune damage to astrocytic AQP4. Recently there is a new group of clinical features with positive AQP4 antibodies which is termed NMO spectrum disorder (NMOSD) with restricted disorders, including recurrent optic neuritis, relapsing transverse myelitis and some encephalitic/brainstem presentations (de Seze and Collongues, 2013). NMOSD is characteristic of positive serum AQP4-antibody and makes it easy to find demyelinating diseases caused by astrocytic impairments more frequent. Alexander disease is another example of how astrocytic dysfunction directly compromises myelination. It occurs ranging from early infanthood to mid-life and causes developmental retardation and seizures. It is a rare non-familial leukodystrophy, primarily caused by mutations in the gene encoding GFAP of astrocytes with associated increases in GFAP, the accumulation of Rosenthal fibers and loss of myelin (Messing et al., 2012). In the process of pathogenesis, normal protoplasmic astrocytes are converted to severe “reactive” astrocytes displaying activation of mTOR cascade, acquirement of CD44, loss of GLT-1 and diminished glutamate transporter current (Sosunov et al., 2013). As such, this indicates that the dysfunctions of astrocytic morphology and physiology make themselves uncoupled to adjacent astrocytes and detrimental to neighboring oligodendrocytes.

Multiple sclerosis (MS) is a representative of primary demyelinating diseases. The hypothesis that MS is primarily caused by astrocytic dysfunction is dated back to 1904. However, their actual influence on MS still remains unclear. In recent decades, mounting evidence shows astrocytes play controversial roles in MS development and pathogenesis. Astrocytes in MS take part in spontaneous myelin repair by secreting trophic factors and providing energy and substrates, such as lactate and iron, to myelin and axons (reviewed by Moore et al., 2011). However, they also contribute to myelin degradation and release pro-inflammatory and inhibitory factors to block this process (Durfinova et al., 2014). In chronic MS lesions, remyelination is absent or mainly in the plaque border because of the formation of hypertrophic astrocytes, scarring fibrous astrocytes or even astrocytic glial scar (Clemente et al., 2013) that inhibit OPCs migration into the lesions thereby preventing their differentiation into mature oligodendrocytes (Bramow et al., 2010). In general, astrocytes in the acute phase are crucial for recovery, while their presence in the chronic phase is inhibitory. Interestingly, MS patients seem to show higher risk of vascular disease compared with those without MS (Durfinova et al., 2014), and are more likely to be hospitalized for ischemic stroke than ischemic heart disease or myocardial infarction (Allen et al., 2008), especially within the first year after a first-time MS diagnosis (Christiansen, 2012). It may be related to endothelial dysfunction whose pathological mechanism is possibly linked to a loss of β2 adrenergic receptors on astrocytes of MS demyelinating lesions (Durfinova et al., 2014).

### Neurodegenerative Diseases

There is a group of distinctive iron-overloaded disorders known as neurodegeneration with brain iron accumulation (NBIA) diseases involved with nine identified disease genes. Among them, aceruloplasminaemia is directly caused by abnormal iron metabolism mainly in astrocytes. It is characteristic of iron excess accumulation caused by gene mutations in the brain and visceral organs, such as liver and pancreas, with a triad of retinal degeneration, diabetes mellitus and brain dysfunction, such as dystonia, ataxia, dementia and depression (Skidmore et al., 2008). Astrocytic ceruloplasmin (CP) is required for iron export from astrocytes to oxidize Fe^2+^ to Fe^3+^, which can bind to extracellular transferring (Figure 1). The functional loss of CP leads to marked iron overload in astrocytes and iron deficiency in surrounding parenchymal cells, for example, oligodendrocytes. Furthermore, iron-overloaded astrocytes also damage ambient parenchymal cells by releasing toxic factors.

Alzheimer’s disease (AD) is the most common form of progressive neurodegeneration in the elderly with the major histopathological hallmarks of extracellular amyloid-β (Aβ) protein accumulation (known as senile plaques) and intracellular neurofibrillary tangles (known as abnormal phosphorylation of tau-protein filaments). At the early stage of AD, astrocytes show atrophy with decreased GFAP-positive area and the reduction in the somatic size and the number of primary processes (Kulijewicz-Nawrot et al., 2012). However, at the late stage of AD, astrogliosis is found widespread with cellular hypertrophy and increases in the number of GFAP and S100β protein (Olabarria et al., 2011). The interaction between astrocytes and Aβ is changeable. Astrocytes contribute to the clearance and degradation of Aβ (Nielsen et al., 2009) and a reduced astrocytic response to Aβ plaques is associated with cognitive impairment, demonstrating relative resistance to Aβ toxicity, at least partly through astrocytic glycolysis (Fu et al., 2015), or on the contrary, astrocytes become an extra source of Aβ (Heneka et al., 2005). It has been reported that Aβ-induced oligodendrocyte apoptosis might be a critical initiating step in AD (Roth et al., 2005). As such, astrocytes have an influence on Aβ metabolism which works in Aβ-induced oligodendrocyte-related AD pathology. This suggests that astrocytes may affect effectively oligodendrocytes *via* Aβ pathways in AD. However, further research in this area is required before definitive conclusions can be drawn.

Amyotrophic lateral sclerosis (ALS) is another “classic” neurodegenerative disorder which is characterized by the progressive loss of corticospinal and spinal motor neurons and involved in progressive reactive astrogliosis and reduction in myelin. ALS is presented with definite white matter pathology, and further researches have shown oligodendrocytes play an early role in ALS, even start to degenerate before motor neurons (Kang et al., 2013a). Oligodendrocyte lineage attempts to compensate for oligodendrocyte loss by increasing their proliferation and differentiation rates (reviewed by Nonneman et al., 2014) but fails because of a loss of MCT1 (Lee et al., 2012), mutant SOD1 or being a direct target of this disease (Stieber et al., 2000). As a kind of cell type with multiplicity and complexity, it is reasonable to speculate astrocytic alterations importantly influence various neuropathological conditions, including ALS. Riluzole, the only FDA-approved drug for ALS, has been found enhancing astrocytic glutamate uptake by up-regulating GLT-1 level and activity (Carbone et al., 2012) and stimulating astrocytic syntheses of NGF, BDNF and GDNF (Mizuta et al., 2001). The ALS model of ablating mutant SOD1 expression selectively in astrocytes doesn’t affect onset, but sharply slows later disease progression (Yamanaka et al., 2008). The evidence indicates damage of oligodendrocyte lineage mainly occurs before or at the onset of ALS, while astrocytes exert their influence during the course of disease.

### Psychiatric Disorders

Mounting literature has displayed the close relation between the lesions of white matter or oligodendrocyte lineage and psychiatric disorders, including schizophrenia, bipolar disorder, depression and autism (Haroutunian et al., 2014). In these diseases, astrocytic changes of physiology and functions have the potential to affect complex behavior and mood. The number of astrocytes in postmortem histological studies of schizophrenia is controversial. Some authors reported no change (Damadzic et al., 2001) while others found increased (Schnieder and Dwork, 2011) or decreased number (Hercher et al., 2014). Fibrillary astrocytes mainly account for the reduced number (Williams et al., 2014), and that the area fraction of GFAP-positive astrocytes decreases and astrocytic spatial distribution demonstrates increased cell clustering in white matter of schizophrenia patients (Hercher et al., 2014). These disparities may be explained, at least in part, by heterogeneity of schizophrenia symptoms and the differences of staining methods and selected markers for assessing the number of astrocytes.

The disturbance of neurotransmission, especially the glutamatergic transmission, may be one of the leading pathological mechanisms of schizophrenia (Laruelle, 2014). As mentioned above, astrocytes are crucial for the sustenance of glutamatergic transmission. Deletion of adenosine A2A receptors in astrocytes disrupts glutamate homeostasis by controlling GLT-1 activity, which is relevant to schizophrenia (Matos et al., 2015). Mutant disrupted-in-schizophrenia 1 (DISC1) in astrocytes reduces the production of D-serine, resulting in compensatory changes in the levels of the amino acid transporters and N-methyl-D-aspartate receptors in the context of tripartite synapse (Abazyan et al., 2014). The EAAT2 expression is reduced in subjects with high-risk metabotropic glutamate receptor 3 haplotype associated with schizophrenia (Spangaro et al., 2012). It is indicated that glutamatergic metabolism in astrocytes may be a potential therapeutic target for schizophrenia.

Actually, many anti-psychiatric drugs take effect through activation of astrocytic pathways but seemingly not including venlafaxine (Zimmermann et al., 2012; Wang J. et al., 2014). Fluoxetine, an anti-depressive drug and one of serotonin-specific reuptake inhibitors (SSRIs), acutely stimulates glycogen synthesis via AKT phosphorylation (Hertz et al., 2014) and also affects glycogenolysis (Xu et al., 2013) in astrocytes. However, chronic treatment with fluoxetine may cause the changes of gene expression in astrocytes (Hertz et al., 2014). Paroxetine, amitriptyline and imipramine affect DNA methyltransferase-1 activity *via* G9a in primary astrocytes (Zimmermann et al., 2012). The chronic treatment with lithium, carbamazepine and valproic acid results in a gradual development of intracellular alkalinization caused by stimulation of acid extruders in astrocytes (Hertz et al., 2014), which alters their functions and may be associated with the fluctuation of pharmacological effects. Altogether, astrocytes are a promising target for the therapy of psychiatric disorders and worthy of further investigations.

## Conclusions and Future Perspectives

It is still poorly understood how astrocytes impact oligodendrocyte lineage and how signals released or expressed by astrocytes affect re-myelination. We have confirmed that reactive astrogliosis is not a simple all-or-none cellular transformation after stimulation based on previous studies. It is a continuum of changes ranging from reversible alterations in gene expression and cell hypertrophy to scar formation with permanent tissue rearrangement both *in vivo* and *in vitro*. These responses are graded, depending on the nature and severity of the insult. After white matter injury, the effect that astrocytes exert on oligodendrocyte lineage is changeable with different reactive conditions, for example, time points, stimuli, signaling pathways represent a few ways for this to occur. Inhibiting specifically detrimental signaling pathways in astrocytes while preserving their beneficial functions may be a promising approach.

Most of the conducted research is concerned with the astrocytic effect on oligodendrocyte lineage or white matter pathology through the use of rodents to mimic human myelin-related disorders. Although some of the research in this area demonstrates hopeful prospects, the reality is that these interventional approaches are not applicable to repairing damaged myelin in the human body. Although the gross morphology of astrocytes is similar to those in rodents, human astrocytes have larger diameter, longer and thicker processes, more GFAP immunoreactions and more complicated synapses, which results in a 16.5-fold increase in astrocytic volume of human brains (Oberheim et al., 2009; Han et al., 2013). In addition, the astrocytic diversity further complicates this issue and leads to the difficulties in interventional pharmacological targeting. It is necessary to consider the heterogeneity of astrocytes and determine the functional implications of astrocytic reactivity in a context-specific manner. Interventions for demyelinating diseases by manipulating astrocytes open new therapeutic vistas and provide potential pharmacological targets.

## Author Contributions

JK and XB provided the idea of this work and designed the structure of the manuscript. JL and LZ wrote the manuscript. YC and BD retrieved and provided much literature. MN and XB revised the manuscript.

## Funding

This work was supported by the National Natural Science Youth Foundation (Project Nos: 30900474 and 81200225).

## Conflict of Interest Statement

The authors declare that the research was conducted in the absence of any commercial or financial relationships that could be construed as a potential conflict of interest.
